# Cognitive efficiency in late midlife is linked to lifestyle characteristics and allostatic load

**DOI:** 10.18632/aging.102243

**Published:** 2019-09-08

**Authors:** Justinas Narbutas, Maxime Van Egroo, Daphne Chylinski, Pamela Villar González, Claudia Garcia Jimenez, Gabriel Besson, Pouya Ghaemmaghami, Grégory Hammad, Vincenzo Muto, Christina Schmidt, André Luxen, Eric Salmon, Pierre Maquet, Christine Bastin, Gilles Vandewalle, Fabienne Collette

**Affiliations:** 1 GIGA-Institute, Cyclotron Research Centre/In Vivo Imaging, University of Liège, Liège 4000, Belgium; 2 Psychology and Neuroscience of Cognition Research Unit, Faculty of Psychology and Educational Sciences, University of Liège, Liège 4000, Belgium; 3 Department of Neurology, CHU Liège, Liège 4000, Belgium

**Keywords:** cognition, midlife, aging, cognitive reserve, allostatic load

## Abstract

We investigated whether cognitive fitness in late midlife is associated with physiological and psychological factors linked to increased risk of age-related cognitive decline. Eighty-one healthy late middle-aged participants (mean age: 59.4 y; range: 50-69 y) were included. Cognitive fitness consisted of a composite score known to be sensitive to early subtle cognitive change. Lifestyle factors (referenced below as cognitive reserve factors; CRF) and affective state were determined through questionnaires, and sleep-wake quality was also assessed through actimetry. Allostatic load (AL) was determined through a large range of objective health measures. Generalized linear mixed models, controlling for sex and age, revealed that higher cognitive reserve and lower allostatic load are related to better cognitive efficiency. Crystallized intelligence, sympathetic nervous system functioning and lipid metabolism were the only sub-fields of CRF and AL to be significantly associated with cognition. These results show that previous lifestyle characteristics and current physiological status are simultaneously explaining variability in cognitive abilities in late midlife. Results further encourage early multimodal prevention programs acting on both of these modifiable factors to preserve cognition during the aging process.

## INTRODUCTION

Cognitive changes associated with normal aging are characterized by a large variability in decline rates and trajectories across cognitive domains [1–4]. Age-related cognitive decline is also highly variable across individuals [5], and inter-individual variability in cognitive changes increases with advancing age [6]. Several physiological and psychological environmental risk and protective factors were proposed to explain this variability [7], such as cognitive and brain reserve [8], affective disorders [9], allostatic load [10], and sleep quality [11].

As proposed by Stern et al. [8], brain reserve is commonly conceived as a neurobiological capital (numbers of neurons, synapses, etc.) while cognitive reserve refers to the adaptability of cognitive processes. These cognitive processes can be influenced by the interaction of innate characteristics (e.g. genetically determined) and cumulative lifetime experiences, that will be the focus of the present study. Contrary to brain reserve, cognitive reserve (CR) is therefore not fixed or immutable. Interestingly, the two kind of reserve help counteracting decline associated with brain aging, pathology or insult ([8]; see also [12] for a complete discussion of the relationships between the concepts of cognitive and brain reserve).

In normal aging, the influence of cognitive reserve factors on cognition is grounded on the positive association between cognitive efficiency and (a) higher level of education and intelligence [13–16], (b) employment complexity and autonomy [17–22], (c) physical activity, engagement in cognitively demanding leisure activities and/or sustained social interactions [23]. These factors also delay pathological cognitive decline, with a later onset of Alzheimer’s disease in individuals with higher reserve [8]. Interestingly, cognitive and brain reserve may have protective and compensatory effects on cognition already in middle-aged people, i.e. around 40 or 50 years old [24].

Among affective disorders, depression and anxiety are the most prevalent in late life [25, 26] and both affect cognition in aging even at a sub-threshold level [27]. High levels of depression and anxiety are associated with decreased performance, particularly for episodic memory and executive functioning [28–32]. Moreover, mild anxiety symptoms in older participants may predict future decline in executive functions [33], while recurrent depression and anxiety in midlife are associated with an increased risk of dementia [34–36].

At the physiological level, several studies emphasized a link between cardiovascular functioning and cognition already in midlife [37–39]. Likewise, lipid and glucose metabolism, inflammation, cortisol level, and sympathetic nervous system functioning are associated with early cognitive decline [38, 40–44]. These physiological measurements were summarized in a comprehensive index of physiological load related to stress, the “allostatic load” [45] that was reported to be negatively associated with episodic memory performance and executive functioning in middle-aged and older adults [10].

Finally, evidence also exists for an influence of sleep quality on cognition in late life [11]. Sleep-wake regulation begins to deteriorate in midlife, and both subjective and objective measures of sleep quality and wakefulness are associated with poorer cognitive fitness, including worse performance in processing speed, memory, and executive function [46–49]. Increased fragmentation of the rest-activity cycle even predicts future cognitive decline and the risk of developing dementia [11, 50].

Some limitations exist in most of these studies, however. Environnemental influences on cognition were mostly considered as independent factors, while they likely compensate or worsen each other [51–53]. In addition, most studies assessed mainly older participants, even though late middle-age (50–70 y.o.) can be considered as a target period as individuals are keeping up with professional engagements despite possible slight (and unnoticed) cognitive decline [54]. They also included a limited evaluation of cognitive efficiency [52, 55], and assessment of physiological factors was sometimes only based on self-report measures [3, 52, 55].

Here, we investigated whether cognition in a group of late middle-aged individuals is associated with cognitive reserve, affective state, allostatic load, and sleep quality. Cognitive status was determined by the Preclinical Alzheimer Cognitive Composite score (PACC5), a composite measure known to be sensitive to early subtle cognitive changes, possibly leading to dementia [56, 57]. Cognitive reserve was measured by assessing educational level, occupational demands, physical activities, and leisure activities across the lifespan. Affective state corresponded to the score on two questionnaires assessing respectively anxiety and depression. Allostatic load was assessed via a comprehensive range of measures: parasympathetic nervous system functioning, cardiovascular functioning, lipid metabolism, glucose metabolism, chronic inflammation, HPA axis functioning, sympathetic nervous system functioning. We evaluated these physiological factors using objective measures, in contrast to some previous studies [3, 52, 55]. Finally, sleep-wake quality was measured based on one objective (actigraphy, measuring the fragmentation of the rest-activity cycle) and two subjective measures (questionnaires assessing sleep quality and daytime sleepiness).

Generalized linear mixed models (GLMM) were applied to compute all statistics. All models included PACC5 as the dependent variable, and controlled for sex and age. At first, GLMM evaluated the association of each global factor (cognitive reserve, affective state, allostatic load, and sleep quality) with PACC5 in separate models. We were also interested in identifying whether specific aspects of each factor were more strongly associated with cognitive status and whether the four aforementioned factors can jointly be related with cognition. Therefore, next statistical models included each component of the global factor (e.g. cognitive reserve was decomposed into its sub-factors), and the final models included the significant predictors of PACC5 that were identified for each factor.

## RESULTS

### Descriptive statistics

Demographics and cognitive outcome (PACC5) are presented in Table 1, while Table 2 gathers raw values of cognitive reserve, affective state, allostatic load, and sleep quality.

**Table 1:** Descriptive statistics of demographical data and cognitive outcome (PACC5) (n = 81).

	**Mean**	**SD**	**Min**	**Max**
**Demographical data**				
Age, *years*	59.41	5.41	50.00	69.00
Sex, *female, n (%)*				54 (66.7 %)
Etnic status, *Caucasian, n (%)*				81 (100 %)
Educational level:				
Primary School				0 (0%)
Secondary School				21 (25.9 %)
Bachelor degree				30 (37.0 %)
Master degree				24 (29.6 %)
PhD or higher				6 (7.4 %)
Socio-economic status*	3.44	0.74	1.00	4.00
**PACC5 (raw scores)**				
FCSRT (0–96)	80.90	6.47	63.00	92.00
Logical Memory Test, *items for delayed recall* (0–25)	12.17	4.00	2.00	22.00
Digit Symbol Substitution Test (0–133)	71.94	12.56	39.00	99.00
Category Fluency, *1 min*	19.79	3.58	10.00	29.00
Mattis Dementia Rating Scale (0–144)	142.38	2.09	134.00	144.00

SD: Standard Deviation; FCSRT: Free and Cued Selective Reminding Test (Free + Total recall).

* Socio-economic status is based on cognitive load charge of the last profession, where 1 means low cognitive load (e.g. office cleaner), and 4 high cognitive load (e.g. secondary schoold teacher).

**Table 2:** Descriptive statistics of raw values of cognitive reserve, affective state, allostatic load, and sleep quality (n = 81).

	**Mean**	**SD**	**Min**	**Max**
**Cognitive reserve**				
Education	15.25	3.11	9.00	25.00
fNART	29.06	4.19	13.00	36.00
Occupation	3815.27	1675.39	22.00	7330.00
Sport	22.31	20.78	.0	111.84
Leisure	3718.43	2393.50	423.78	11173.31
**Affective state**				
Beck Depression Inventory	5.26	4.50	.0	17.00
Beck Anxiety Inventory	3.10	3.33	.0	17.00
**Allostatic load**				
*Cardiovascular functioning*				
Systolic blood pressure, *mm Hg*	119.07	11.70	92.50	150.00
Heart rate, *bpm*	60.38	9.12	42.40	84.50
Pulse pressure, *mm Hg*	45.44	10.02	25.00	65.00
*Parasympathetic nervous system functioning*				
SDANN, *ms* (higher is better)	54.32	39.08	13.00	224.00
RMSSD, *ms* (higher is better)	59.41	60.45	7.00	324.00
*Lipid metabolism*				
Body mass index, *kg/m^2^*	24.78	2.84	19.37	30.12
Waist-hips ratio, *cm/cm*	.99	.17	.67	1.37
LDL, *mg/dL*	134.08	32.40	45.00	237.00
HDL, *mg/dL* (higher is better)	66.17	18.94	28.00	149.00
Triglycerides, *mg/dL*	109.83	54.31	29.00	339.20
*Glucose metabolism*				
Glycated hemoglobin, *%*	5.39	.29	4.40	6.30
Fasting blood sugar, *mg/dL*	90.88	11.65	72.00	129.00
*Chronic inflammation*				
C-reactive protein, *mg/L*	1.90	2.16	.16	9.50
Interleukin-6, *pg/ml*	2.91	5.29	.70	38.00
*HPA axis functioning*				
DHEA-S, *μmol/L*	3.39	1.75	.42	10.57
Urine cortisol, *μg/24h*	48.07	23.60	14.29	138.75
*Sympathetic nervous system functioning*				
Urine adrenaline, *μg/24h*	9.43	4.39	3.06	25.47
Urine noradrenaline, *μg/24h*	40.34	15.46	11.22	95.89
**Sleep quality**				
Intradaily variability	.43	.12	.26	.96
PSQI	4.85	2.94	.0	13.00
ESS	6.16	4.04	.0	16.00

SD: standard deviation; fNART: National Adult Reading Test French version; SDANN: standard deviation of average heart beat-to-beat intervals; RMSSD: root mean square of successive differences between normal heartbeats; LDL: fasting blood low-density lipoprotein cholesterol; HDL: fasting blood high-density lipoprotein cholesterol; HPA: hypothalamic-pituitary-adrenal; DHEA-S: fasting blood dehydroepiandrosterone sulfate; PSQI: Pittsburgh Sleep Quality Index; ESS: Epworth Sleepiness Scale (see Methods section for the references of the tests and questionnaires).

### Effect of cognitive reserve

**Figure 1:**
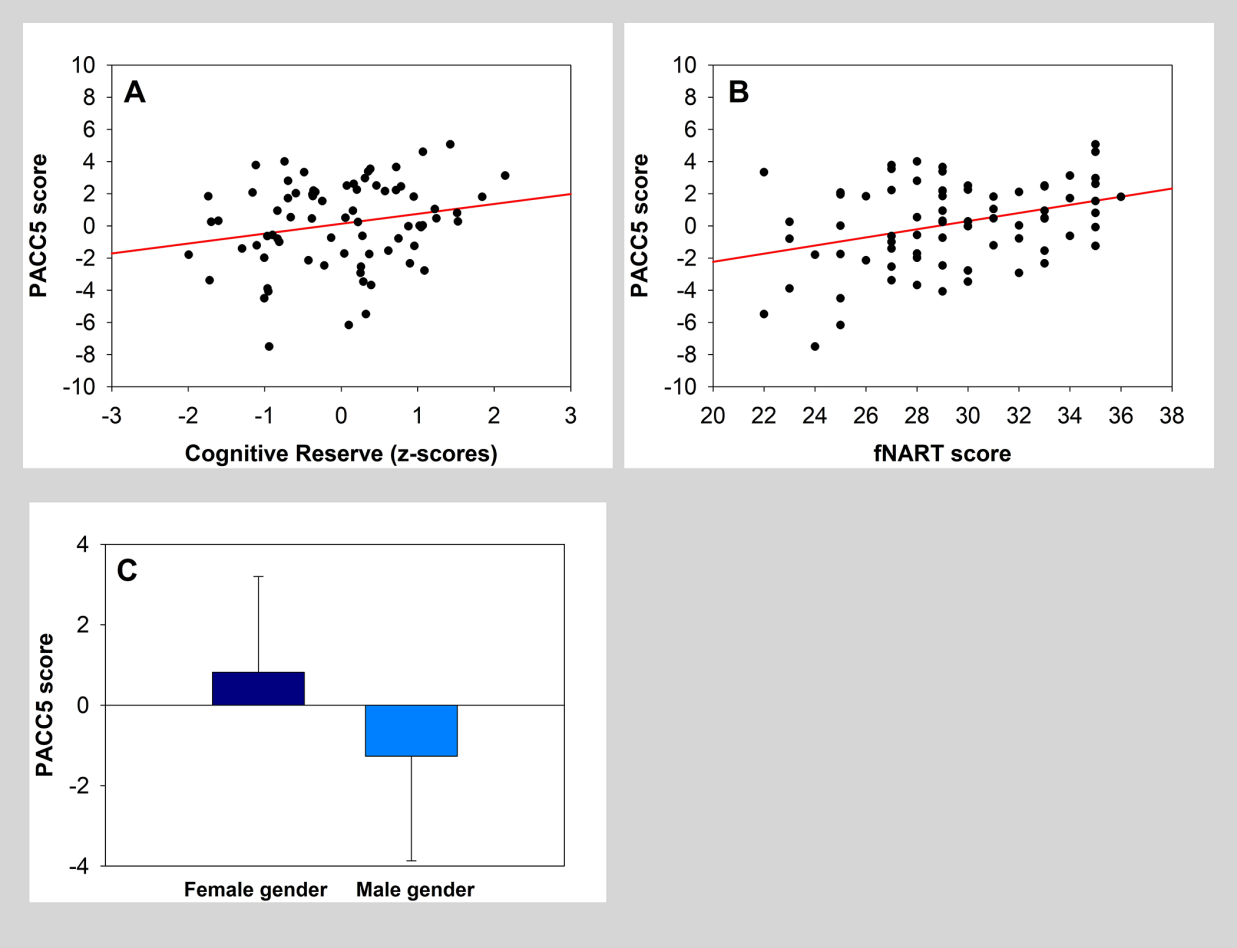
(**A**, **B**) Scatter plots visualizing the association between PACC5 and cognitive reserve measures (global measure and fNART). Regressions were used for visual display only, and not as a substitute for the full GLMM statistics presented in Table 3. (**C**) Bar plot visualizing PACC5 score according to sex.

Global measure of cognitive reserve was significantly positively associated with PACC5 performance (Table 3, grey area and Figure 1A). Assessment of the association between PACC5 and all separate measures of cognitive reserve (Table 3, white area) showed a significant positive association with the score of the French version National Adult Reading Test (fNART), a proxy of crystallized intelligence (Table 3; Figure 1B). Moreover, sex was also significantly associated with PACC5 in the two models, with a better performance for women (Figure 1C).

**Table 3:** Statistical outcome of the GLMM seeking for associations between PACC5 (dependent variable) and global cognitive reserve, and its sub-scores (n = 75; 5 outliers removed, one missing data).

	**Estimate ± SE**	**F value (df)**	**P**
Sex	−2.24 ± .59	14.58 (1,71)	**.0003** (R_sp_^2^=.17)
Age	−.47 ± .28	2.93 (1,71)	.09
Cognitive Reserve (global)	.87 ± .29	8.64 (1,71)	**.004** (R_sp_^2^=.11)
Sex	−1.97 ± .65	9.05 (1,67)	**.004** (R_sp_^2^=.12)
Age	−.45 ± .29	2.41 (1,67)	.13
Education	−.12 ± .34	.12 (1,67)	.73
fNART	1.02 ± .34	8.89 (1,67)	**.004** (R_sp_^2^=.12)
Occupation	−.05 ± .31	.03 (1,67)	.86
Sport	.14 ± .39	.14 (1,67)	.71
Leisure	.10 ± .34	.09 (1,67)	.77

Grey and white parts represent distinct models. Rsp^2^: Semi-partial R^2^, SE: Standard Error; df: degrees of freedom; fNART: National Adult Reading Test (French version).

### Effect of affective state

GLMM analysis showed that global score of affective state was not significantly associated with PACC5 performance (Table 4, grey area). We then evaluated the impact of the two separate affective measures, depression and anxiety, on PACC5 score (Table 4, white area). None of those measures were found to be significantly associated with PACC5 performance. As in the previous model, sex was significantly associated with PACC5, both in analyses with global factor and with specific sub-scores.

**Table 4:** Statistical outcome of the GLMM seeking for associations between PACC5 (dependent variable) and global affective state, and its sub-scores (n = 80; one outlier removed).

	**Estimate ± SE**	**F value (df)**	**P**
Sex	−1.81 ± .66	7.57 (1,76)	**.007** (R_sp_^2^=.09)
Age	−.15 ± .30	.25 (1,76)	.62
Affective State (global)	.11 ± .33	.11 (1,76)	.74
Sex	−1.81 ± .66	7.44 (1,75)	**.008** (R_sp_^2^=.09)
Age	−.15 ± .31	.23 (1,75)	.64
Depression	.05 ± .36	.02 (1,75)	.90
Anxiety	.08 ± .39	.04 (1,75)	.84

Grey and white parts represent distinct models. Rsp^2^: Semi-partial R^2^; SE: Standard Error; df: degrees of freedom.

### Effect of allostatic load

**Figure 2:**
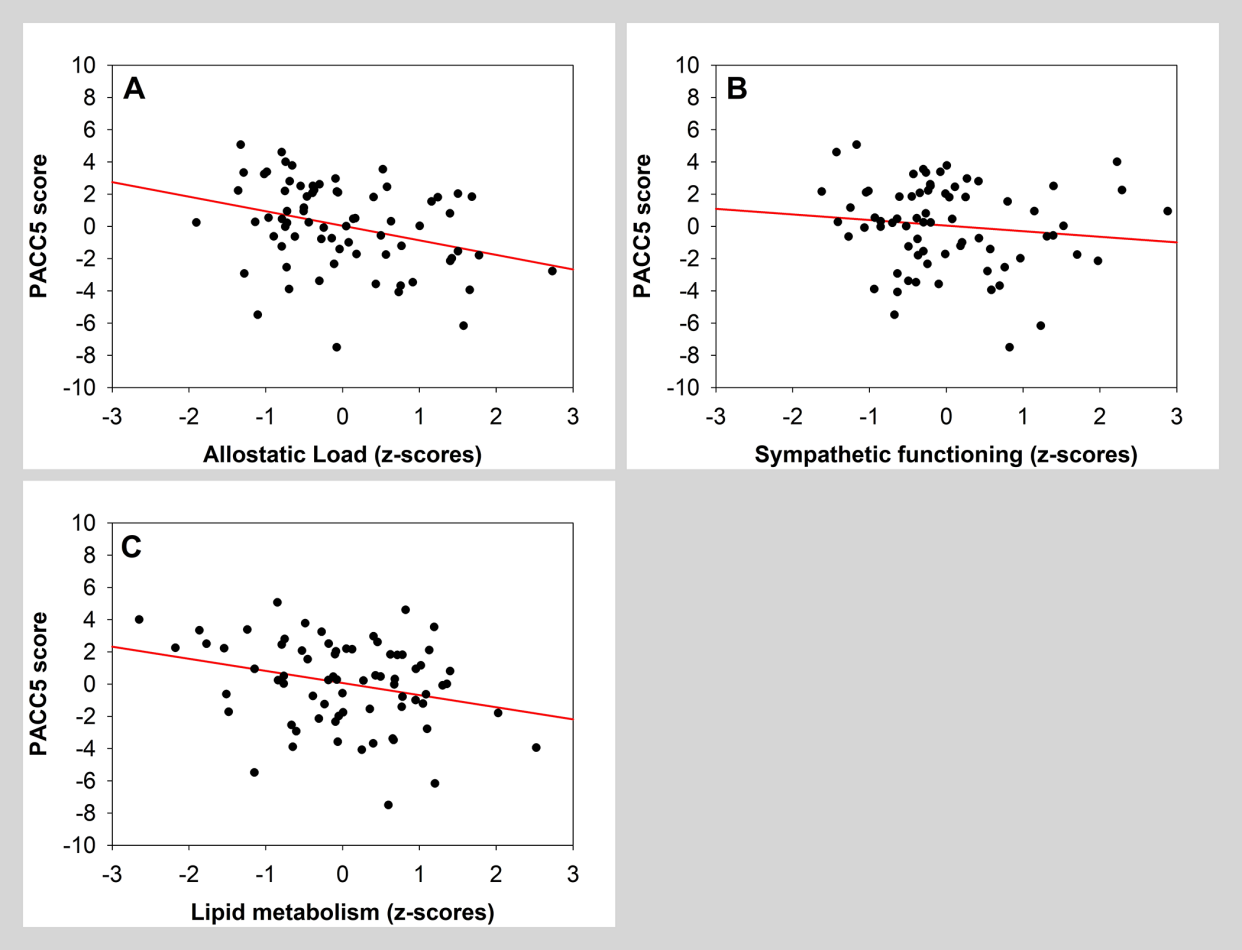
**Scatter plots visualizing association between PACC5 and measures of allostatic load (global measure, sympathetic functioning and lipid metabolism).** Regressions were used for visual display only, and not as a substitute of the full GLMM statistics presented in Table 5.

GLMM analysis revealed that global measure of allostatic load was significantly negatively associated with PACC5 score (Table 5, grey area; Figure 2A). A separate analysis further indicated that, among all factors composing allostatic load, both sympathetic functioning and lipid metabolism were significantly and negatively associated with PACC5 (Table 5, white area; Figure 2B and 2C). Sex was significantly associated with PACC5 in the model with the global allostatic load measure.

**Table 5:** Statistical outcome of the GLMM seeking for associations between PACC5 (dependent variable) and global allostatic load, and its sub-scores (n = 72; 8 outliers removed; one missing data).

	**Estimate ± SE**	**F value (df)**	**P**
Sex	−1.50 ± .62	5.84 (1,68)	**.03** (R_sp_^2^=.08)
Age	−.16 ± .30	.29 (1,68)	.59
Allostatic Load (global)	−.71 ± .32	5.02 (1,68)	**.03** (R_sp_^2^=.07)
Sex	−1.18 ± .64	3.38 (1,62)	.07
Age	−.57 ± .32	3.15 (1,62)	.08
Cardiovascular functioning	.60 ± .34	3.17 (1,62)	.08
Parasympathetic functioning	−.77 ± .40	3.67 (1,62)	.06
Lipid metabolism	−.87 ± .36	5.86 (1,62)	**.02** (R_sp_^2^=.09)
Glucose metabolism	−.06 ± .40	0.03 (1,62)	.87
Chronic inflammation	−.23 ± .57	0.16 (1,62)	.69
HPA axis functioning	−.23 ± .31	0.55 (1,62)	.46
Sympathetic functioning	−.93 ± .35	7.03 (1,62)	**.01** (R_sp_^2^=.10)

Grey and white parts represent distinct models. Rsp^2^: Semi-partial R^2^; SE: Standard Error; df: degrees of freedom; HPA: hypothalamic-pituitary-adrenal.

### Effect of sleep-wake quality

Global measure of sleep-wake quality was not significantly associated with PACC5 (Table 6, grey area). Considering sleep factors separately in a second model, they did not yield any significant associations with PACC5 (Table 6, white area). Sex was significantly associated with PACC5 in both models.

**Table 6:** Statistical outcome of the GLMM seeking for associations between PACC5 (dependent variable) and global sleep quality, and its sub-scores (n = 80; one outlier removed).

	**Estimate ± SE**	**F value (df)**	**P**
Sex	−1.69 ± .63	7.24 (1,76)	**.009** (R_sp_^2^=.09)
Age	−.18 ± .30	.36 (1,76)	.55
Sleep Quality (global)	−.002 ± .31	.0 (1,76)	.99
Sex	−1.67 ± .64	6.75 (1,74)	**.01** (R_sp_^2^=.08)
Age	−.16 ± .34	.23 (1,74)	.63
IV	−.03 ± .39	.01 (1,74)	.93
PSQI	−.03 ± .30	.01 (1,74)	.92
ESS	−.05 ± .30	.02 (1,74)	.88

Grey and white parts represent distinct models. Rsp^2^: Semi-partial R^2^; SE: Standard Error; df: degrees of freedom; IV: Intradaily variability; PSQI: Pittsburgh Sleep Quality Index; ESS: Epworth Sleepiness Scale.

### Simultaneous effect of cognitive reserve and allostatic load

In a final set of models, we included the global factors (cognitive reserve and allostatic load) or sub-factors (fNART, sympathetic functioning and lipid metabolism) that were significantly associated with PACC5 in the previous models. The analysis on global factors revealed that while cognitive reserve was positively associated with PACC5, allostatic load had no significant association (Table 7, grey area). When fNART, sympathetic functioning and lipid metabolism were included in a same model, fNART was significantly and positively associated with PACC5, and both sympathetic functioning and lipid metabolism were significantly negatively associated with the cognitive score (Table 7, white area).

**Table 7:** Statistical outcome of the GLMM seeking for associations between PACC5 (dependent variable), cognitive reserve, allostatic load, and their sub-scores that were significant in previous models (n = 67; 12 outliers removed; 2 missing data).

	**Estimate ± SE**	**F value (df)**	**P**
Sex	−2.09 ± .65	10.36 (1,62)	**.002** (R_sp_^2^=.14)
Age	−.29 ± .30	.93 (1,62)	.34
Cognitive Reserve (global)	.74 ± .33	5.01 (1,62)	**.03** (R_sp_^2^=.07)
Allostatic Load (global)	−.44 ± .32	1.85 (1,62)	.18
Sex	−1.58 ± .60	6.99 (1,61)	**.01** (R_sp_^2^=.10)
Age	−.46 ± .28	2.68 (1,61)	.11
fNART	1.09 ± .31	12.01 (1,61)	**.001** (R_sp_^2^=.16)
Sympathetic functioning	−.65 ± .31	4.40 (1,61)	**.04** (R_sp_^2^=.07)
Lipid metabolism	−.73 ± .31	5.42 (1,61)	**.02** (R_sp_^2^=.08)

Grey and white parts represent distinct models. Rsp^2^: Semi-partial R^2^, SE: Standard Error; df: degrees of freedom, fNART: National Adult Reading Test (French version).

## DISCUSSION

This study investigated how cognitive performance in late middle-aged individuals is associated with cognitive reserve, affective state, allostatic load, and sleep quality taken separately and simultaneously. Our main finding is that, in late middle-age, higher cognitive reserve and lower allostatic load are related to better cognitive efficiency when assessed through the PACC5 composite score. This is observed when the two global measures are considered apart. Our results further show that the only sub-factors to be significantly associated with cognition were crystallized intelligence in CR factors, and both sympathetic nervous system functioning and lipid metabolism in AL. Moreover, when these three specific measures were considered together, they all remained significantly associated with PACC5-derived cognitive efficiency. Compared to the other measures we considered, these three measures stand therefore as the most associated with cognitive efficiency in late middle age.

A positive influence of specific aspects of cognitive reserve on cognition was previously reported in older individuals [17, 18, 20, 51, 52]. We have demonstrated here that this effect can be observed already in late midlife. Moreover, the most significant contribution to cognitive reserve in our middle-aged population was that of the fNART score, pointing to a predominant protective role of crystallized intelligence in accordance with previous studies [15, 58, 59]. Crystallized intelligence, which refers to specific, acquired knowledge (e.g. vocabulary, general information), has been shown to be even a better predictor of cognitive efficiency in aging than education [15, 59].

Interestingly, Richards and Sacker suggested that crystallized intelligence in middle-age is independently determined at first by childhood cognition, subsequently by educational level, and finally by occupational attainment [60]. Therefore, it seems that innate characteristics, i.e. genetic, are a main determinant of cognitive reserve [61, 62]. Based on these data, we can suggest that, in our sample, other lifestyle characteristics contribute much less to maintaining cognitive abilities as they are mainly driven by crystallized intelligence (fNART performance) determining educational level that itself increases cognitive reserve by fostering the development of new cognitive strategies [59]. Moreover, 69.5% of our participants were still professionally active, implying that the respective contribution of occupational demands and leisure (intellectual and physical) activities on cognitive reserve varied substantially in our sample [17]. Our sample may therefore still be too small to efficiently grasp such a “noisy” profile and track other expected links with cognitive efficiency. Beyond the scope of this study, some recent data suggest that epigenetic traits, which can boost or interefere with the transcription of specific genes, could also influence the relationships between life environment and cognitive efficiency. Indeed, epigenetic traits are heritable [63], and are affected by chemical exposure, medication, and lifestyle (e.g. diet) [64]. Therefore, it would be interesting to include genetic and epigenetic measures (e.g. methylation) and to assess parental lifestyle in future studies focusing on the link between environmental factors and late-life cognition.

Aside from cognitive reserve, our data confirmed that a global score of allostatic load is significantly associated with global cognition in late midlife [10, 45]. Our sub-factor analyses revealed that sympathetic functioning and lipid metabolism seem to be important factors for global cognition in midlife. The measurement of sympathetic functioning was based on 24-h urinary adrenaline and noradrenaline excretion, the latter being the main sympathetic neurotransmitter in circulatory regulation [65]. Elevated levels of stress increase sympathetic arousal, which, in turn, impairs working memory and cognitive flexibility [66]. Similar processes may drive the association detected here. With regard to lipd metabolism, it was showed previously that high levels of total cholesterol and low-density lipoprotein (LDL) cholesterol, were associated with cognitive decline in older population [44], and that low-level of high-density lipoprotein (HDL) cholesterol is a risk factor for deficit and decline in memory in midlife [67, 68]. HDL cholesterol is critical for the maturation of synapses and maintenance of synaptic plasticity while LDL cholesterol is a proven risk factor for cardiovascular disease. Interestingly, our data showed that a global measure of lipid metabolism (including body mass index and waist-to-hip circumference ratio) may already emphasize the negative effect of a high lipidic load on cognition in late middle-age. Finally, several studies showed that cardiovascular functioning is among the best physiological predictors of cognitive efficiency [10, 37, 38]. However, this is not what we have observed in our sub-factor analysis (with a tendency for higher level of cardiovascular issues related to better cognition), probably because cardiovascular measures (i.e. systolic blood pressure, heart rate) rarely exceeded normative cut-points in our sample [10], thus limiting inter-individual variability.

Importantly, the specific measures of crystallized intelligence, sympathetic nervous functioning and lipid metabolism jointly explained cognitive efficiency (with the larger explanatory power for the former) while the association between global allostatic load factor and PACC5 was no longer present when the global effect of cognitive reserve was taken into account. This suggest that both global factors collectively contribute to cognitive efficiency, but with a lower explanatory power for allostatic load. Based on these results, we suggest that the global measure of allostatic load is partly determined by the level of cognitive reserve. Indeed, some lifestyle characteristics leading to high cognitive reserve (e.g. sociocultural level, educational level, employement, etc.) could establish living standards that promote low allostatic load (e.g. easy access to medical care, healthy food, low air pollution, etc.) [69, 70]. Another explanation could be that the variability of allostatic load in our sample is restricted in comparison to variability in cognitive reserve as we included participants with relatively good health status (i.e. no smokers, no sleep disorders, no excessive alcohol consumption, no diabetes, etc.), and exclusion criteria were unlikely to have repercussions on the measurement of cognitive reserve. Consequently, allostatic load could have a lower explanatory power due to an indirect selection bias.

We observed no associations between affective state or sleep-wake quality and cognitive efficiency, in contrast to several previous studies [28, 32, 46–49]. This suggests that, in our sample, affective and sleep-wake dimensions are less associated with PACC5 cognitive efficiency relative to CR and AL. We excluded volunteers with ongoing pharmacological treatment for depression or anxiety or with moderate to high levels of depression or anxiety according to established questionnaires. Moreover, we evaluated current affective state, while it may be the cumulative effect of chronic depression and anxiety that have more important negative impact on cognition [35, 71]. Consequently, future research should include lifetime affective dimensions. Furthermore, the type of sleep-wake measures we included, i.e. subjective perception of sleep and wakefulness quality, and actigraphy to quantify rest-activity cycle fragmentation [72], may explain the absence of association with cognitive efficiency in our sample. We did not include electroencephalography and/or polysomnography in the analyses which may provide a more refined phenotype. In addition, participants with sleep apnea were excluded, and our sample was relatively young and healthy. The high level of cognitive reserve of our sample may also prevent subtle differences in cognition to be associated with coarse measures of sleep and wakefulness quality. Previous associations between rest-activity fragmentation and cognition were detected in larger and older sample size that were not screened for sleep disorders (e.g. N = 144, age = 69.5 ± 8.5 in [46]; N = 737, age = 81.6 ± 7.2 in [50]. In addition, even though our analyses suggest that no direct interaction between parameters could explain our findings (see Methods section), complex or indirect interactions between sleep-wake quality or affective dimensions and either CR or AL may still exist (e.g. anxiety may affect sleep quality which in turn may affect AL).

We also observed an unexpected sex effect, with women performing better than men in most of our statistical models, and these results cannot be explained by differences in factors such as age, education, tendencies for depression or anxiety, or subjective cognitive complaints (see Table 8). Presence of sex differences on multi-compound scores was sometimes reported in the literature [73], but not systematically [56]. Proposed mechanisms to explain sex effect might involve hormonal differences [74], genetic factors, differences in brain networks, socioeconomic roles, and health choices [75].

**Table 8:** T-test comparisons of socio-demographic characteristics, depression, anxiety, dementia scale, subjective cognitive complaints, and vocabulary level according to sex in study sample (n = 81): mean ± standard deviation.

	**Male gender (n = 27)**	**Female gender (n = 54)**	**P**
Age	60.56 ± 5.54	58.83 ± 5.31	.18
Education, *years*	15.89 ± 3.71	14.93 ± 2.74	.19
Depression (BDI)	4.04 ± 3.28	5.87 ± 4.92	**.05**
Anxiety (BAI)	2.70 ± 4.22	3.35 ± 2.76	.47
Mattis Dementia Rating Scale	142.20 ± 2.20	142.50 ± 2.04	.55
Subjective cognitive complaints (CDS)	28.41 ± 19.95	28.17 ± 19.57	.96
Vocabulary level (fNART)	29.41 ± 4.14	28.89 ± 4.25	.60

BDI: Beck Depression Inventory [80]; BAI: Beck Anxiety Inventory [81]; CDS: Cognitive Difficulties Scale [100]; fNART: National Adult Reading Test (French version).

The main aim of this study was to assess whether factors known to influence cognitive fitness in aging would be associated with cognitive performance in late middle-aged individuals. We demonstrated that global measures of cognitive reserve and allostatic load significantly explain cognitive efficiency in our participants. In our late middle-aged healthy and cognitively normal sample devoid of sleep apnea, these associations were stronger than potential undetected links between PACC5 scores and affective status and sleep-wake quality measured through questionnaires and actigraphy. These results can be discussed in the context of the revised model of *Scaffolding Theory of Aging and Cognition* (STAC-r, proposed by Reuter-Lorenz & Park [76]) that combines a life-span and a life-course approach to understand and predict cognitive status and rate of cognitive change over time. Indeed, the model proposes that low global allostatic load decreases the negative influence of physiological stress on brain structures and functions, potentially leading to increased possibility of preservation of neural resources (brain maintenance, see [76]). Furthermore, a high level of cognitive reserve might have a positive influence both on the build-up of cognitive compensatory strategies, and also on the implementation of brain compensatory networks [76]. The STAC-r model also states that the two factors can act independently on cognition, as shown by the absence of interactive effects in our statistical models. Finally, we have observed a larger impact of cognitive reserve than allostatic load (see R_sp_^2^ in Table 7). As previously discussed, the influence of cognitive reserve is here mainly driven by crystallized intelligence that would lead to better socio-economic level (see [60]), and consequently, to a lifestyle with lower allostatic load [77]. Our protocol did not assess how brain neurotransmitters could affect the effect of cognitive reserve on cognition so we can only speculate about potential mechanisms. For example, cholinergic neurotransmission may be involved as it was associated with brain plasticity and showed to affect episodic memory performance in healthy older individuals [78], and correlated with proxies of cognitive reserve (education and occupation) in prodromal and early stages of Alzheimer’s disease [79].

This study provided information on psychological and physiological mechanisms influencing cognition in aging people free of major comorbidities or health issues. These results should be replicated by including participants with unhealthy lifestyles (high alcohol consumption, smoking, or drug habits), or with common age-related health issues (obesity, diabetes, untreated hypertension or sleep apnea, etc.). This should allow to generalize the significance of this study and hopefully resolve some contradictory findings observed in previous papers [29, 32, 37, 38, 46–48].

In conclusion, our results indicate that allostatic load and cognitive reserve most strongly predict cognitive efficiency in healthy late middle-age. Interestingly, these factors are modifiable [7], thus we can act on both across the whole lifespan by following a healthy way of life (e.g. health monitoring, stress management, Mediterranean diet, etc.) and promoting a cognitively stimulating environment (e.g., acquiring new knowledge or abilities, social and intellectual leisure activities, etc.) [76]. Future research should therefore implement multimodal prevention program in middle-age population at risk for dementia, in order to confirm that improvement of these factors allow the preservation of cognitive efficiency, and maybe prevent or delay dementia in later life.

## MATERIALS AND METHODS

**Participants** were healthy late middle-aged (50 to 69 y.o.) French speaking men and women (Table 1; N=81; 54 women [66.7%]). No participants reported any recent history of neurological or psychiatric disease, or were taking medication likely to affect the central nervous system. All had normal or corrected-to-normal vision and hearing. Other exclusion criteria were sleep apnea/hypopnea index ≥ 15/h, assessed during an in-lab night of sleep under polysomnography, body mass index < 18 and > 29 kg/m², smoking, illicit drug consumption, excessive consumption of caffeine (> 4 cups/day) or alcohol (> 14 units/week), diabetes, and shift-work. Participants with high levels of depression and anxiety as assessed by the Beck Depression Inventory [80] and by the 21-items self-rated Beck Anxiety Inventory [81], respectively, were excluded (i.e. score > 17), as well as participants with clinical level of depression or anxiety with ongoing pharmacological treatment. Moreover, participants with treated (> 6 months) hypertension and hypothyroidism were included. All participants showed normal performance on the Mattis Dementia Rating Scale [82] [i.e. score > 130], eliminating individuals with neuropsychological evidence of cognitive impairment. The experimental procedures were approved by the Local Ethics Committee of the Faculty of Medicine (University of Liege). All participants gave their signed informed consent prior to the experiment and received a financial compensation.

**Neuropsychological examination** consisted of a battery of cognitive tasks assessing short-term and episodic memory, attentional and executive functions. Original Preclinical Alzheimer’s Cognitive Composite 5 (PACC5) score [56, 57] was computed as the sum of z-scores of the following cognitive measures: *Free and Total Recall in the Free and Cued Selective Reminding Test (FCSRT)* [83], Delayed Recall in the *Logical Memory Test* [84], Total score in the *Digit Symbol Substitution Test* [85], scores in the *Verbal Fluency Test* for the categories of Animals, Fruits and Vegetables (1 min each), and *Mini Mental State Examination* [86]. Here we introduced three changes to the initial PACC5: Mini Mental State Examination was replaced by the score from the Mattis Dementia Rating Scale, the Verbal Fluency Test score was calculated for the animal category only, and a more recent version of the Digit Symbol Substitution Test having a larger range of scores was used [87].

Neuropsychological evaluation was performed during 2 sessions taking approximately 75 min each. As our study was designed for several research objectives, additional neuropsychological tasks were included in the assessment, which are not mentioned here.

**Cognitive reserve** was determined based on a computerized version of lifestyle questionnaire [88] assessing educational level, occupational demands, physical activities, and leisure activities across the lifespan.

*Educational level* was calculated as the number of completed *years of formal education*.

*Crystallized intelligence* was assessed as the total score at the National Adult Reading Test French version (fNART) [89].

*Occupational demands’ score* was calculated as the level and the duration of cognitive load associated with work experience during lifespan, and is expressed by the following formula: $Occupation=\sum_{i=1}^{n}\left(c_{i}\times h_{i}\times y_{i}\right)$, where: *c_i_* represents cognitive load classified according to the International Standard Classification of Occupations from the International Labour Organization [90, 91]; *h_i_* represents the number of hours per week for the occupation; *y_i_* – the number of years at a job; *n* – number of occupations across the lifespan.

*Physical activity’s score* was calculated as Metabolic Equivalent of Task (MET) per week over the lifespan [92]. Self-report of any regular physical activities since age 12 were collected together with their estimated intensity (low or high), number of years of practice, number of months per year, and number of hours per week. MET was computed as follows: $P_{X}=\sum_{i=1}^{5}\left(MET_{i}\times t_{i}\right)$, where: *P_X_* – life period (4 different periods); *MET_i_* – MET of one physical activity; *t_i_* –hours per week. Thus, the global *physical activity’s score* is the average MET per week during 4 life periods (12–18 y.o., 19–34 y.o., 35–49 y.o., and after 50 y.o.).

*Leisure activities’ score* was based on being engaged or not in 9 leisure activities since age 6: reading books, reading journals, domestic activities, mental solitary activity, cultural activity, artistic activity, volunteering, social activity, and social games. For each activity, participants had to indicate life periods of practice, frequency, and time interval (e.g. from 6 to 12 y.o., once per week). Separate *leisure activities’ scores* were calculated as follows: $L_{i}=\sum_{i=1}^{n}\left(f_{i}\times t_{i}\times p_{i}\right)$, where: *L_i_* – one type of leisure activity; *n* – number of life periods; *f_i_* – frequency of leisure activity (e.g. playing social game *one time*); *t_i_* – time interval of leisure activity (e.g. playing social game *f_i_* times *a week*); *p_i_* – life period (number of years). Activities not practiced at all were scored 0. Thus, the global *leisure activities’ score* is the average of the scores for the 9 leisure activities.

Global score of cognitive reserve was computed as a z-score of the average of all z-scored measures. Higher values in all cognitive reserve measures mean better cognitive reserve.

**Affective state** was measured as the averaged z-scores of two questionnaires collected after one of the neuropsychological evaluation sessions, the Beck Depression Inventory (BDI) [80], and the Beck Anxiety Inventory (BAI) [81]. Higher values mean worse affective state.

**Allostatic load** was assessed via a comprehensive range of measures [10]. The global score of allostatic load was calculated as the z-score of the average of its 7 sub-scores presented below. Each of seven sub-scores was also calculated as the z-score of the average of the constituting measures. Each participant underwent an electrocardiography (ECG) recording via 2 sub-clavicular bipolar electrodes using Embla N7000 amplifier (Natus, Pleasanton, USA) during 5 min at rest in semirecumbent position in the evening (app. 1h prior to habitual sleep time). Fasting blood sample was also collected from each participant upon awakening in the morning. Moreover, 24-hour urine collection was also carried out in the lab.

*Parasympathetic nervous system functioning* was calculated based on 2 ECG measures computed by Embla RemLogic software (Natus, Pleasanton, USA): standard deviation of the average heart beat-to-beat intervals (SDANN), and root mean square of successive differences between normal heartbeats (RMSSD).

*Cardiovascular functioning* was calculated based on measures of systolic blood pressure, heart rate during ECG recordings, and pulse pressure (difference between the systolic and diastolic pressure at rest).

*Lipid metabolism* was calculated based on measures of body mass index (BMI), waist-hip ratio and blood measures of low-density lipoprotein cholesterol (LDL), high-density lipoprotein cholesterol (HDL), and triglycerides.

*Glucose metabolism* was calculated based on blood measures of glycated hemoglobin (HbA1C) level and glucose level.

*Chronic inflammation* was calculated based on blood measures of C-reactive protein level and interleukin-6 level.

*HPA axis functioning* was calculated based on measures of blood dehydroepiandrosterone sulfate (DHEA-S), and urinary 24-hour excretion of cortisol. Cortisol excretion was corrected for serum creatinine level to adjust for lean body mass [10].

*Sympathetic nervous system functioning* was calculated based on the following urinary measures: 24-hour excretion of adrenaline, and 24-hour excretion of noradrenaline. Both adrenaline and noradrenaline excretion were also corrected for serum creatinine level to adjust for lean body mass [10].

Higher raw values in physiological measures mean higher allostatic load, to the exception of HDL, DHEA-S, SDANN, and RMSSD values. Consequently, the signs of HDL, DHEA-S, SDANN and RMSSD were reversed when calculating respectively lipid metabolism, HPA axis functioning, and parasympathetic nervous system functioning. Therefore, higher values in the global score of allostatic load and its sub-scores mean higher allostatic load.

**Sleep-wake quality** was measured based one objective (actigraphy) and two subjective measures (questionnaires).

*Intradaily variability* (IV), which measures the fragmentation of the rest-activity cycle, and is a proxy of sleep-wake fragmentation [93], was obtained from actigraphy data (Actiwatch AW4, CamNtech Ltd., Cambridge, UK) collected during 14 days, without any instruction with respect to sleep and wake. We used in-house software for automatic actigraphy scoring (pyActigraphy v0.1; DOI: http://doi.org/10.5281/zenodo.2537921). Periods of inactivity exceeding 120 min were excluded from the analysis. Actigraphy data was hourly clustered, epoch length defined as 1 min, and activity threshold set at 4 per min for subsequent data binarization. Finally, intradaily variability [94] was calculated using the following formula: *IV = c^1h^/d^1h^*, with: $d^{1h}=\sum_{i}^{n}\frac{{\left(x_{i}-\bar{x}\right)}^{2}}{n}$, and with: $c^{1h}=\sum_{i}^{n-1}\frac{{\left(x_{i+1}-x_{i}\right)}^{2}}{n-1}\;$, where *x_i_* is the number of active minutes during the *i^th^* period, $\bar{x}$ is the mean of all data, and *n* is the number of periods covered by the actigraphy data. Higher IV reflects more fragmented rest-activity cycle.

*Subjective sleep quality* was evaluated using the Pittsburgh Sleep Quality Index (PSQI [95]) Higher values on PSQI show higher degree of sleep disturbances.

*Excessive daytime sleepiness* was assessed using Epworth Sleepiness Scale (ESS [96]), with higher values indicating higher daytime sleepiness.

Overall sleep-wake quality was computed as the z-score of averaged z-scores of the three aforementioned factors, with higher value reflecting worse sleep-wake quality.

### Statistical analyses

All statistical analyses were performed with SAS 9.4 for Windows (SAS Institute, Cary, USA). Generalized linear mixed models (GLMM; PROC GLIMMIX) were applied to compute all statistics following the determination of the distribution of dependent variables using ‘allfitdist’ function on MATLAB R2013a (MathWorks Inc., Natick, USA). In all GLMM, collinearity diagnosis was performed on all predictors using Tolerance (TOL) and Variance Inflation Factors (VIF) as criteria. Degrees of freedom (DF) were estimated using Kenward-Roger’s correction. Subject (intercept) effect was included as a random factor. We defined *p-value* < 0.05 as significant. Data points situated ± 3 SD from their mean were defined as outliers and removed. As a result, sample size ranged from 67 to 80 across models; each of the 81 participants contributed to at least 6 models in Tables 3 to 7.

All models included PACC5 as the dependent variable, and controlled for sex and age. At first, GLMM evaluated the association of each global factor (cognitive reserve, affective state, allostatic load, and sleep quality) with PACC5 in separate models. Then, models included each component of the global factor (e.g. cognitive reserve was decomposed into its sub-factors). The final models included the significant predictors of PACC5 that were identified for each factor. Comparison of Bayesian Information Criterion (BIC) values for models with and without interactive term showed better explanatory power when interaction effects were not modelled. Consequently, only models without interaction term were analysed. Semi-partial R^2^ (R_sp_^2^) was reported for each significant effect as described previously [97], provided that DF are estimated using Kenward-Roger’s methods. Due to outlier values (± 3 SD), DF and sample size vary from one model to another (from N = 67 to N = 80).

We estimated the sensitivity of our analyses using G*Power 3.1.9.4 software taking into account our sample size (n = 81), a power of .8, type I error probability = .05 [98, 99] in a one t-tailed linear multiple regression model including sex and age as predictor. This analyses indicated that the minimal effect size we could detect was r = .28 (R^2^ = .08).
